# Mean Heart Dose Prediction Using Parameters of Single-Slice Computed Tomography and Body Mass Index: Machine Learning Approach for Radiotherapy of Left-Sided Breast Cancer of Asian Patients

**DOI:** 10.3390/curroncol30080537

**Published:** 2023-08-04

**Authors:** Wlla E. Al-Hammad, Masahiro Kuroda, Ryo Kamizaki, Nouha Tekiki, Hinata Ishizaka, Kazuhiro Kuroda, Kohei Sugimoto, Masataka Oita, Yoshinori Tanabe, Majd Barham, Irfan Sugianto, Yudai Shimizu, Yuki Nakamitsu, Junichi Asaumi

**Affiliations:** 1 Department of Oral and Maxillofacial Radiology, Okayama University Graduate School of Medicine, Dentistry and Pharmaceutical Sciences, Okayama 700-8558, Japan; wealhammad@just.edu.jo (W.E.A.-H.);; 2 Department of Oral Medicine and Oral Surgery, Faculty of Dentistry, Jordan University of Science and Technology, Irbid 22110, Jordan; 3 Radiological Technology, Graduate School of Health Sciences, Okayama University, Okayama 700-8558, Japan; 4 Department of Health and Welfare Science, Graduate School of Health and Welfare Science, Okayama Prefectural University, Okayama 719-1197, Japan; 5 Graduate School of Interdisciplinary Sciences and Engineering in Health Systems, Okayama University, Okayama 770-8558, Japan; 6 Department of Dentistry and Dental Surgery, College of Medicine and Health Sciences, An-Najah National University, Nablus 44839, Palestine; 7 Department of Oral Radiology, Faculty of Dentistry, Hasanuddin University, Sulawesi 90245, Indonesia

**Keywords:** breast cancer, radiotherapy, heart dose, machine learning, deep neural network, deep inspiration breath-hold technique, computed tomography

## Abstract

Deep inspiration breath-hold (DIBH) is an excellent technique to reduce the incidental radiation received by the heart during radiotherapy in patients with breast cancer. However, DIBH is costly and time-consuming for patients and radiotherapy staff. In Asian countries, the use of DIBH is restricted due to the limited number of patients with a high mean heart dose (MHD) and the shortage of radiotherapy personnel and equipment compared to that in the USA. This study aimed to develop, evaluate, and compare the performance of ten machine learning algorithms for predicting MHD using a patient’s body mass index and single-slice CT parameters to identify patients who may not require DIBH. Machine learning models were built and tested using a dataset containing 207 patients with left-sided breast cancer who were treated with field-in-field radiotherapy with free breathing. The average MHD was 251 cGy. Stratified repeated four-fold cross-validation was used to build models using 165 training data. The models were compared internally using their average performance metrics: F2 score, AUC, recall, accuracy, Cohen’s kappa, and Matthews correlation coefficient. The final performance evaluation for each model was further externally analyzed using 42 unseen test data. The performance of each model was evaluated as a binary classifier by setting the cut-off value of MHD ≥ 300 cGy. The deep neural network (DNN) achieved the highest F2 score (78.9%). Most models successfully classified all patients with high MHD as true positive. This study indicates that the ten models, especially the DNN, might have the potential to identify patients who may not require DIBH.

## 1. Introduction

Radiotherapy (RT) plays an important role in the treatment of breast cancer [1,2]. However, RT has late adverse effects on the heart, which can significantly influence patient survival rates [3,4]. Generally, it has been found that these late effects can be detected within 10 years after RT [1]. Previous cardiac toxicity studies analyzed radiation exposure to the whole heart using dosimetric variables such as mean heart dose (MHD) [4,5,6]. These historical studies have assessed the MHD as a significant measure of radiation exposure. Therefore, researchers are now aiming to minimize MHD in order to improve the overall survival rate.

Deep inspiration breath-hold (DIBH) is a technique for reducing MHD in patients with left-sided breast cancer and is commonly used worldwide [7,8,9]. In this technique, patients are instructed to take a deep breath before treatment and hold it throughout the delivery of radiation; consequently, the lungs fill with air, and the heart is displaced from the treatment area, resulting in a lower radiation dose to the heart compared to the free-breathing (FB) technique. However, the DIBH technique is costly and time-consuming for patients and RT staff [10].

In Asian countries, the shortage of RT personnel and equipment, as well as the limitations of the health insurance system, reduce the utilization of the DIBH technique in comparison to the USA [11,12]. In general, the breast volume of Asian patients is much lower than that of American or European patients, and as MHD is greatly affected by breast volume, the use of DIBH may be preferable for Asian patients with large breast volumes [13]. Furthermore, it has been reported that the number of Asian patients who received high MHD was much lower than those who received low MHD, which indicates that not all Asian patients require DIBH [13]. Thus, it is desirable to select Asian patients who may not require DIBH before RT planning for left-sided breast cancer. Thus, time and cost can be saved for most Asian patients by avoiding the need for double planning for FB and DIBH radiotherapies.

Recently, artificial intelligence (AI) and machine learning (ML) techniques have been frequently implemented in the field of RT [14,15,16]. However, there are limited studies on utilizing ML to predict MHD using clinical and radiographic parameters. It is important to note that AI-based techniques can be proved or disproved using some performance metrics or formal verification methods, including safety, fault tolerance, fairness, robustness, and correctness [17]. To the best of our knowledge, no previous study has evaluated the potential role of ML algorithms in the selection of patients that may not need DIBH.

This study aimed to develop various ML models for predicting MHD and identifying patients with left-sided breast cancer who may not need DIBH in Asian countries.

## 2. Materials and Methods

### 2.1. Dataset and Study Setting

This study included 207 females with early-stage left-sided breast cancer who received radiation therapy with FB at Okayama University Hospital between 2009 and 2016. The patients had breast cancer with stages of 0–II according to the tumor–node–metastasis-based staging of breast cancers (8th ed.) by the Union for International Cancer Control. A total of 38 patients had stage 0, 118 patients had stage I, and 51 patients had stage II breast cancer. The patients had a mean age of 55.3 ± 11.1 years (age range: 31–78 years). All patients were irradiated for whole breast with 200 cGy per fraction, 25 fractions, for a total of 5000 cGy, after partial breast resection. Patients were treated in our hospital using either the conventional field-in-field (FIF) with one reference point (FIF-1RP) method or a new FIF with two reference points (FIF-2RP) method [18]. Eighty-eight patients were irradiated with an additional 1000–1600 cGy boost on the tumor bed. The heart dose during the 5000 cGy irradiation was the subject of this study [13]. 

Patients provided written informed consent for undergoing RT and using their anonymous data for scientific studies. This study was conducted in accordance with the Declaration of Helsinki, as revised in 2013. The Ethical Review Board of our institute approved the use of anonymous post-radiation therapy data for this study (approval no. 2103-024).

**Figure 1:**
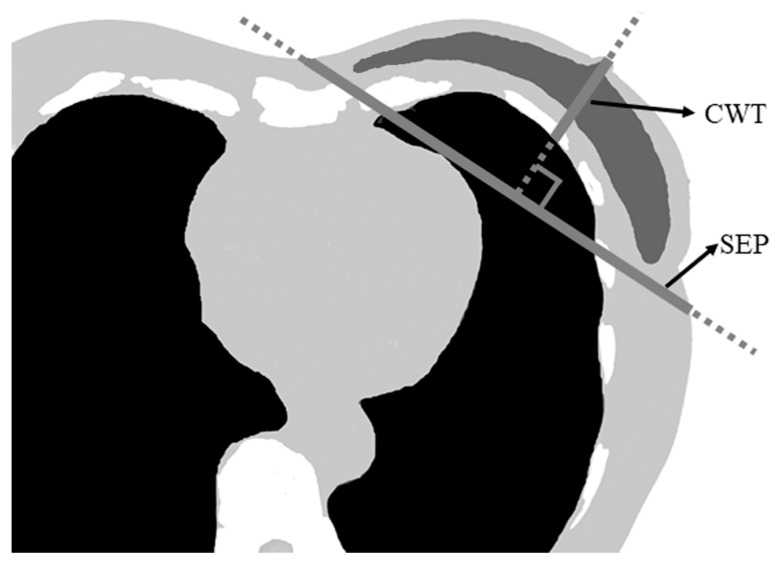
Single-slice CT parameters. Separation was defined as the distance along the posterior edge of the tangent fields at the nipple level. Chest wall thickness was defined as the distance from the nipple surface to the lung on a perpendicular line of breast separation. CWT—chest wall thickness; SEP—breast separation.

In March 2021, the following data were retrospectively collected from the radiation treatment planning system after CT simulation: breast separation (SEP), chest wall thickness (CWT), and MHD. The SEP and CWT were measured for each patient using single-slice CT obtained at the nipple level, as shown in Figure 1. SEP was defined as the distance along the posterior edge of the tangent fields, while CWT was defined as the distance from the nipple surface to the lung on a perpendicular line of the SEP. Additionally, the following data were collected from the clinical records of each patient: age, body mass index (BMI), tumor site, and radiation treatment method. The dataset was analyzed using the statistical software SPSS version 27 (IBM Corp., Armonk, NY, USA) to test the correlation between the collected data and MHD, with the statistical methods used being the Spearman and Eta correlation coefficients. The correlation coefficient values and the *p*-values for statistical significance are shown in Table 1. The variables that had a significant correlation with MHD were identified and considered as independent variables for ML. Three variables were ultimately selected as independent variables for ML: BMI, CWT, and SEP. MHD was considered a dependent variable.

In this study, Anaconda Python version 3.9 and Python libraries (Python Software Foundation, Wilmington, Delaware, USA) were utilized for building, developing, and experimenting with our ML models. We used an imbalanced dataset that consisted of 207 breast cancer patients, with 165 patients of low MHD (<300 cGy) and 42 patients of high MHD (≥300 cGy) [6,13,18]. The average MHD was 251 cGy. Furthermore, we utilized the synthetic minority over-sampling technique (SMOTE) along with the ‘imblearn’ pipeline to increase the number of high-MHD patients in the training dataset [19].

### 2.2. ML Algorithms

ML algorithms are procedures that are implemented in code and run on data. In this study, we utilized ten supervised ML algorithms to accurately classify patients into low or high MHD. These ML algorithms were used to model the relationships and dependencies between MHD and the three selected variables, enabling us to predict whether MHD is low or high for new data based on the relationships learned from our previous dataset.

The ML algorithms used in this study were logistic regression (LR), decision tree (DT), K-nearest neighbors (KNN), naïve Bayes (NB), ridge classifier (RC), support vector machine (SVM), bagging, gradient boosting (GB), random forest (RF), and deep neural network (DNN). 

### 2.3. Models Building

**Figure 2:**
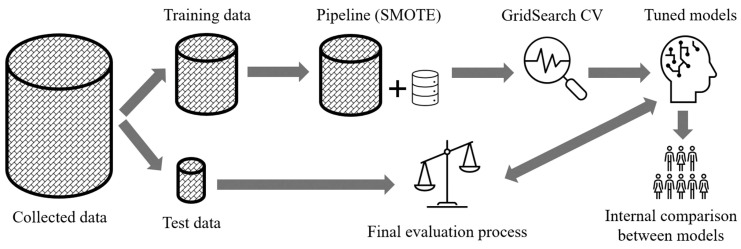
Overview of criteria of building models. SMOTE—synthetic minority oversampling technique; CV—cross-validation.

Every ML algorithm generates its unique ML model. A general overview of the building model criteria is shown in Figure 2. The initial step was to randomly split the dataset into training and test datasets using a ratio of 80:20. Considering that we used a highly imbalanced dataset, it was necessary to split the dataset using a stratified train–test split. This method was well-suited to the nature of our dataset and split it in a way that preserved the same proportions of patients in each class (low MHD, high MHD) as observed in the original dataset, with 80% of each class in the training dataset and 20% of each class in the test dataset [20].

The second step was to use a training dataset to select the best parameters for each model in a process called hyperparameter tuning. In this study, our objective was to accurately predict the patients who might not need DIBH, with a focus on minimizing false negatives (false low MHD). Therefore, the models were trained using the F2 score with a grid search cross-validation (GridSearchCV). The F2 score was determined as the primary metric to compare the performance of the models. 

**Figure 3:**
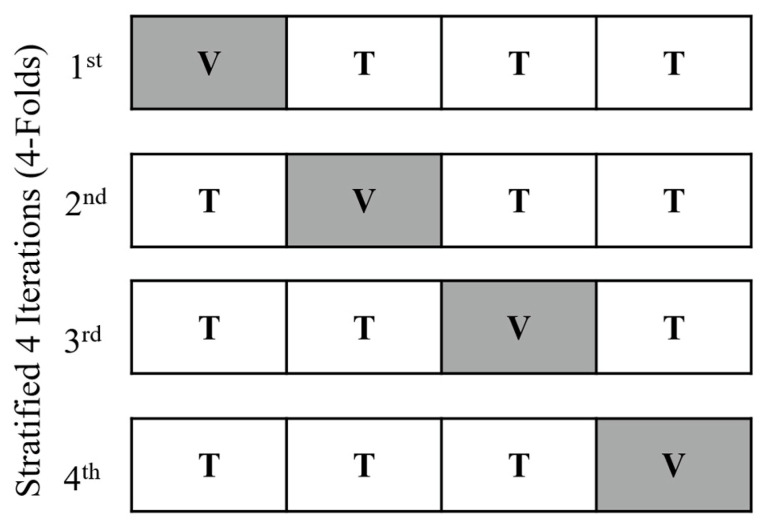
The process of stratified 4-fold cross-validation. The dataset is randomly split into 4 stratified folds. Each fold is used as a validation set once (the shaded area), while the other folds are temporarily combined to form a training set for model generation. Performance metrics on the validation set are calculated and stored, and the process is repeated for 4 iterations. This process of stratified 4-fold cross-validation is repeated 5 times. T—training fold; V—validation fold.

The third step involved building the models along with the optimal values of the hyperparameters for each model. To avoid overfitting during the model evaluation process, we used repeated stratified 4-fold cross-validation (CV). The process of stratified 4-fold CV is shown in Figure 3. During this process, the training dataset was randomly divided into 4 folds, each with approximately the same distribution for each class, where 3 folds were used as the training set to develop the ML models while the remaining fold was used as the validation set to estimate the performance of the models internally within the training dataset. Along with the process of CV, the new synthetic high-MHD patients resulting from SMOTE were only added in the training folds and not the validation folds by using the ‘imblearn’ pipeline. This was performed to ensure that our models were validated using only real data. After 4 iterations and each validation fold was used exactly once, we repeated the whole CV process 5 times using repeated stratified 4-fold CV, with a different division of the training dataset each time. Finally, comparisons between the models were performed internally using the averaged performance metrics generated from the CV process. The averaged performance metrics were the following: F2 score, AUC, recall, accuracy, Cohen’s kappa, and Matthews correlation coefficient (MCC).

### 2.4. Models Performance Testing

Although CV is intended to prevent overfitting when developing predictive models, external validation through a reserved testing dataset is necessary to evaluate their prediction performance [16]. Therefore, the model performance was further evaluated externally using data from 42 patients who were not used for training or building the models. The average MHD of the testing dataset was 250 cGy and contained 9 patients of high MHD and 33 patients of low MHD. The classification cut-off value was set as MHD ≥ 300 cGy. Each model’s performance was computed using the following performance metrics: F2 score, AUC, recall, accuracy, Cohen’s kappa, and MCC. In this study, the classification success index was not computed because of the use of an imbalanced dataset.

The supplementary file (File S1) provides further information on what these metrics represent.

## 3. Results

### 3.1. Hyperparameter Tuning

Table 2 shows the results of the hyperparameter tuning process, which selects the optimal values for each hyperparameter in our model. These values were used for building and training each ML model.

### 3.2. Internal Comparison between Models

The internally averaged predictive performance of each ML model within the CV process is listed in Table 3. Two models, KNN (66.5%) and GB (65.5%), achieved slightly higher F2 score values than the others. For the AUC, GB and KNN again achieved higher score values than the other models (72.4% and 72.2%, respectively).

KNN had the highest recall score (85%), followed by SVM (79.5%). In terms of accuracy, bagging (69.6%) and GB (68.5%) achieved the highest scores.

For Cohen’s kappa, GB and bagging achieved higher score values than the other models (0.342 and 0.320, respectively). Whereas for MCC, KNN, GB, and bagging achieved higher score values than the other models (0.382, 0.364, and 0.352, respectively). 

**Figure 4:**
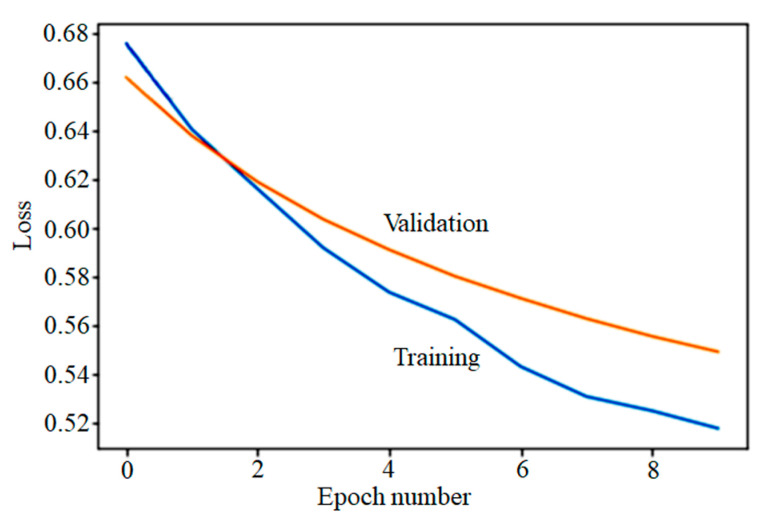
The influence of the dataset’s characteristics on the credibility of internal cross-validation results in deep neural network. A dropout layer was used with a rate of 0.1. The optimization method was Adam. Binary cross-entropy was used as a loss function with a sigmoid activation for the output layer.

Figure 4 shows the loss for training and validation per epoch in the DNN model.

### 3.3. Final Evaluation of Model Performances 

The final predictive performances of our models are presented in Table 4. Each model was evaluated using an external test dataset (n = 42).

Most of our models correctly predicted all high-MHD patients in the test dataset, resulting in a recall score of 100% for most models. However, three models misclassified one high-MHD patient as low MHD: NB, RC, and LR.

DNN achieved the best performance among all models, with an F2 score of 78.9%, an AUC score value of 81.8%, a recall score of 100%, an accuracy score of 71.4%, a Cohen’s kappa score of 0.428, and an MCC of 0.522.

**Figure 5:**
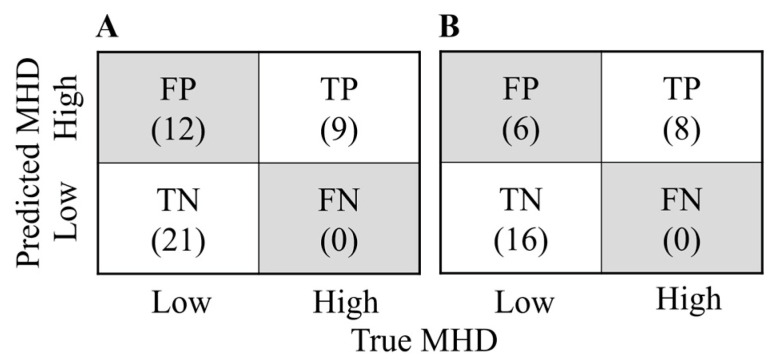
Confusion matrix of DNN model. (**A**) Confusion matrix of DNN prediction of whole dataset patients. (**B**) Confusion matrix of DNN prediction of only FIF-2RP patients. FP—false positive; TP—true positive; TN—true negative; FN—false negative; MHD—mean heart dose.

Additionally, we sub-analyzed DNN performance in the classification of high-MHD patients treated by the FIF-2RP method. In this case, the DNN achieved an F2 score of 86.9%, an AUC score of 86.3%, a recall score of 100%, and an accuracy score of 80%. The prediction summary generated by the DNN is shown as a confusion matrix in Figure 5.

## 4. Discussion

In this study, we used ten ML algorithms to develop models for the prediction of MHD based on patient radiographic and clinical factors to identify those who might not require DIBH. The predictive performances of the models were evaluated and compared. Based on their final evaluation results, the DNN algorithm achieved the best performance, with an F2 score of 78.9% and an AUC score of 81.8%.

MHD is known to have a significant correlation with late cardiac toxicity during breast RT [3,4,5,6]. Therefore, it is strongly recommended to establish methods to reduce MHD [4,5,6]. The International Quantitative Analysis of Normal Tissue Effects in the Clinic (QUANTEC) guidelines stated that radiation-induced cardiac death at 10 years is high if MHD is more than 300 cGy [6]. For that reason, in this study, the classification cut-off value was set as MHD ≥ 300 cGy. The FIF-2RP method has been shown to significantly reduce skin dose and slightly reduce MHD in patients with breast cancer [17]. Ishizaka et al. reported that during FIF RT, MHD is increased by increasing the treated breast volume [13]. Therefore, to predict MHD in our AI study, we included input variables related to breast volume, such as SEP and CWT.

In recent years, AI and ML have already been widely implemented in radiation oncology, particularly in treatment planning, segmentation, radiation physics, quality assurance, and contouring or image-guided RT [14,16]. However, the use of this technology by itself, especially in clinical settings, is still limited [14]. Prior studies have predicted MHD using AI and ML approaches with the aim of selecting patients with a potential risk for cardiac toxicity [21,22,23,24] and reducing it by performing the DIBH technique [21,24]. In most of these studies, MHD prediction was dependent on CT parameters such as maximum heart distance or cardiac contact distance [24]. The prediction of MHD in these studies requires significant time and effort, as it is typically performed after full CT simulation. Whereas, in this study, the prediction was performed using single-slice CT simulation, which enables a faster and easier prediction process. In a study of 94 left-sided breast cancer patients, Koide et al. suggested the potential of using a convolutional neural network (CNN) to generate affected CT scans by breath-hold, resulting in high performance and well-visualized prediction of ΔMHD (AUC = 99.5%), which equals the MHD reduction between the FB and DIBH techniques. However, the prediction is a time-consuming process, as planning a CT session for DIBH requires an additional 30 min for each patient than undergoing CT with the FB technique [21]. In contrast, Koide et al. also reported that non-CT parameters are promising as predictors of MHD in FB technique, as demonstrated by their use of a CNN based on preoperative chest X-rays of 103 patients with left-sided breast cancer (AUC = 86.4%) [24]. In both studies, Koide et al. attempted to predict ΔMHD, rather than MHD, on FB using the CNN; meanwhile, the performance of the models was evaluated by setting the cut-off value of ∆MHD >100 cGy. In our study, the cut-off value of the classification was set as MHD ≥ 300 cGy. To the best of our knowledge, no previous study has evaluated the performance of MHD prediction using the absolute cut-off value of MHD.

Few studies have predicted MHD using clinical parameters such as age, BMI, and pulmonary function tests [9,25,26]. Predictions using clinical parameters may have some advantages in terms of early availability and reduced patient radiation exposure; however, these reports do not have a high prediction performance in comparison with using radiologic parameters, such as chest X-rays or CT parameters. Yamauchi et al. suggested a possible relationship between BMI and the feasibility of DIBH, indicating that the degree of benefit from DIBH varies with BMI [9]. Therefore, in our study, we combined both clinical (BMI) and radiographic (single-slice CT parameters) parameters to achieve the best prediction of MHD, considering the significant role of BMI and the early timing of the prediction using single-slice CT parameters, which can be acquired more easily than whole CT slice parameters. To the best of our knowledge, this study is the first to predict MHD using single-slice CT to select Asian patients who might not require DIBH.

ML utilizes programmed algorithms to analyze input data and make predictions within an acceptable range. These algorithms learn and improve their predictions by optimizing themselves based on the data they receive. When new data is introduced, the algorithms tend to make increasingly precise predictions. ML algorithms can be classified into three main categories based on their purposes and the method used to teach the underlying machine: supervised, unsupervised, and semi-supervised. In this study, we utilized supervised ML algorithms. Supervised ML algorithms start by utilizing a labeled training dataset to train the underlying algorithm. Subsequently, this trained algorithm is employed to categorize an unlabeled test dataset into similar groups. Supervised learning algorithms suit well with classification problems [27].

LR is an established and good method used for supervised classification. However, as shown by our results, LR was easily outperformed by other algorithms. The major limitation of LR is the assumption of linearity between the dependent variable and the independent variables. Non-linear problems, such as our classification problem, cannot be solved with LR because it has a linear decision surface. Moreover, linearly separable data is rarely found in real-world scenarios. Thus, it is tough to obtain complex relationships using LR [27,28].

DT, NB, and RC are simple algorithms that have low computational requirements, resulting in shorter implementation times and lower performance scores. On the other hand, RF, KNN, GB, and SVM are more sophisticated algorithms that necessitate a significant amount of processing time. While these algorithms provide accurate and precise results, they are not easily interpretable [27,28].

As for the predictive power of algorithms, DNNs and CNNs, a special type of DNN, are best known for image-related prediction [21,22,24]. However, some authors have reported that DNN and CNN were not the best when comparing the predictive power of many algorithms [29,30]. Hou et al. reported that extreme GB was the best among different algorithms, including DNN, for predicting the incidence of breast cancer in 7127 Chinese women using 10 breast cancer risk factors, suggesting that this might be due to the use of a low-dimensional dataset, which means using few independent variables in relation to the large dataset they used [29]. Deist et al. also reported that RF and elastic net logistic regression achieved the best predictive performance among DNN and other algorithms in predicting RT outcomes and toxicity risk among 3496 patients based on clinical, dosimetric, and blood biomarker features from multiple institutions [30].

Although our results showed a discrepancy between internal CV and external validation results, previous studies have reported that such discrepancies might occur, especially when using a small dataset [31,32]. The internal CV process effectively uses limited data, and the evaluation results might closely approximate the performance of the model on the test set [31]. However, Bleeker et al. confirmed that for relatively small datasets, internal validation results are not sufficient to determine the model’s usability for future settings. Figure 4 shows the loss for training and validation per epoch in the DNN model. In small imbalanced datasets, a single wrong prediction with confidence will drop performance metrics slightly, but the loss will still increase. Therefore, a substantial external dataset carries greater significance and is commonly regarded as providing a population that is more comparable [32]. 

FIF-2RP is a recently developed FIF radiation technique, particularly suitable for patients with relatively small breast volumes. In such patients, high-dose areas remain in the irradiation field around the reference point in conventional FIF-1RP, whereas the development of the FIF-2RP technique in our institution has improved dosimetric parameters, which led to a reduction in high-dose ranges and subsequent skin toxicities [18]. Tekiki et al. reported that the FIF-2RP method decreased the high-dose range V105% of the target to 0% while maintaining a homogeneous dose distribution across the breast tissue. This decrease in the high-dose range was in conjunction with a decrease in the occurrence and grade of skin adverse events. Therefore, the FIF-2RP could be advised as an optimal method in clinical practice for patients with early-stage breast cancer [18]. In this study, the predictive power of the DNN using FIF-2RP patients alone was analyzed on an external test dataset, indicating that this DNN model might be suitable for predicting MHD with the FIF-2RP technique.

**Figure 6:**
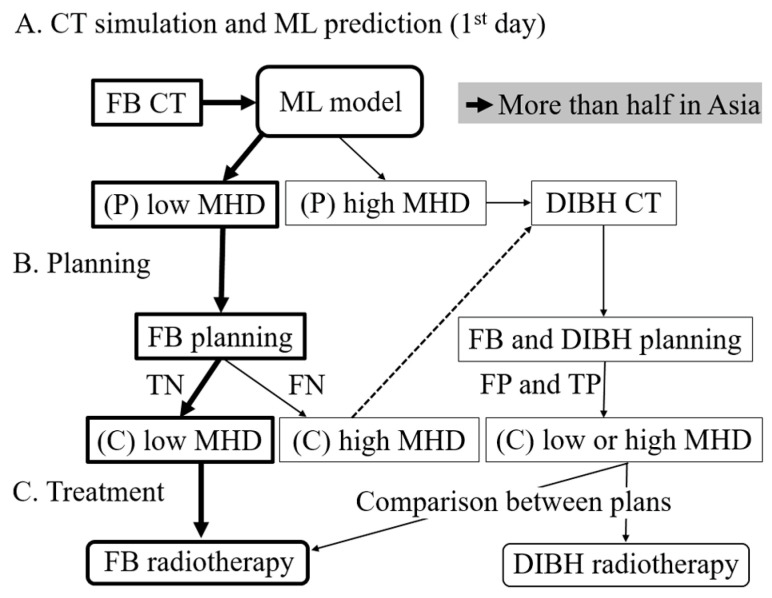
The role of earlier prediction of MHD using machine learning models in choosing the suitable breast radiotherapy planning. (**A**) On the first day of the patient’s visit to the radiotherapy institution, the simulation of free-breathing CT and estimation of the patient’s mean heart dose through a machine learning model will be performed. Bold arrow indicates patients whose number might be more than half in Asian countries. (**B**) The radiotherapy planning process for free-breathing and deep inspiration breath-hold techniques will then be carried out. (**C**) A final decision on the most suitable radiotherapy treatment will be reached. FB—free-breathing; ML—machine learning; P—predicted; MHD—mean heart dose; DIBH—deep inspiration breath-hold; TN—true negative; FN—false negative; FP—false positive; TP—true positive; C—calculated.

Our study offers a possible clinical application for the models prior to RT planning for left-sided breast cancer in Asian patients. Figure 6 indicates the role of earlier prediction of MHD using our model in selecting suitable breast RT planning for patients. As per our models, if the predicted MHD for a patient is low, it is recommended to consider planning for FB RT. The oncologist will be prepared to proceed with FB planning. However, if it is determined that the patient would receive a high MHD with the FB planning, a CT scan with the DIBH technique should be conducted, and the planning process for DIBH will be initiated. At this stage, the oncologist will compare the MHD between the FB and DIBH plans. If there is a significant difference in the MHD between the two plans, DIBH RT will be chosen. If the difference is not significant, then FB RT will be selected. It should be emphasized that the majority of Asian patients, over half of them, exhibit low MHD. Our results demonstrate that the models effectively classify low MHD by reducing the likelihood of misclassifying high MHD as a false negative. Consequently, these models will significantly assist oncologists in selecting appropriate planning options at an earlier stage. As a result, time and cost can be saved for most Asian patients by avoiding the need for double planning for FB and DIBH radiotherapies.

This study has several limitations. First, our datasets consisted of a small number of patients. However, previous studies on the prediction of MHD also used datasets between 60 and 209 patients [21,22,23,24]. Second, we encountered a challenge due to the highly imbalanced dataset that we used, which only included a few high-MHD patients in both the training and test datasets. This made it difficult to achieve maximum performance for our models. Third, our study used a single institutional dataset, similar to most previous studies on MHD prediction. However, multi-center input data should be used to improve model generalizability [22]. Therefore, future studies are required to build models using larger and more balanced datasets from multiple centers to ensure the generalizability of the models. Fourth, our study was only conducted and limited to those facilities which routinely use the DIBH technique.

## 5. Conclusions

In conclusion, our study has shown that all ten ML algorithms achieved good results in identifying MHD in patients with left-sided breast cancer using clinical and single-slice CT parameters. The DNN model was the best choice for the prediction of MHD and might be useful for identifying patients that may not need DIBH. The findings of this study may contribute to the daily clinical practice of RT for breast cancer treatment.

## Figures and Tables

**Table 1:** Patient characteristics.

Variable	Characteristics	Correlation Coefficient	*p*-Value
Body mass index (median (IQR))	22.2 (20.2–25.2)	0.408 ^a^	<0.001
Chest wall thickness (median (IQR), cm)	6 (5.1–6.8)	0.335 ^a^	<0.001
Separation (median (IQR), cm)	19 (17.1–20.2)	0.290 ^a^	<0.001
Tumor site (%)		0.134 ^b^	0.456
Upper-inner quadrant	27.1%		
Lower-inner quadrant	9.2%		
Upper-outer quadrant	51.7%		
Lower-outer quadrant	4.8%		
Central portion	7.2%		
Radiation method; n		0.054 ^b^	0.452
FIF-1RP	70		
FIF-2RP	137		
Age (median (IQR), years)	56 (46–64)	0.006 ^a^	0.926

IQR: interquartile range. Correlation coefficient and its *p*-value between mean heart dose and each variable were calculated using ^a^ Spearman’s correlation coefficient (rs) and ^b^ Eta correlation ratio (η). Body mass index was calculated as weight (kg)/height^2^ (m). Chest wall thickness (cm) was defined as the distance from the skin surface to the lung at the nipple level. Separation (cm) was defined as the distance along the posterior edge of the tangent fields at the nipple level. Tumor site was defined according to the International Classification of Diseases for Oncology (third edition). Radiation method (n) was the number of patients treated using either field-in-field with one reference point or field-in-field with two reference points.

**Table 2:** Hyperparameter tuning results.

Machine Learning Algorithm	Hyperparameter Name	Best Value
Deep Neural Network	batch_size	32
	dropout	0.1
	epoch	10
	optimizer	“adam”
	activation	“relu”, “sigmoid”
	init	“uniform”
	dense_nparams	256
Random Forest	max_depth	2
	max_features	“sqrt”
	min_samples_split	2
	n_estimators	5
K-Nearest Neighbors	metric	“euclidean”
	n_neighbors	37
	weights	“uniform”
Bagging	max_samples	0.1
	n_estimators	37
Gradient Boosting	learning_rate	0.001
	max_depth	2
	n_estimators	15
	subsample	0.1
Support Vector Machine	C	0.11
	gamma	“scale”
	kernel	“rbf”
Decision Tree	max_depth	1
	min_samples_split	2
Naïve Bayes	alpha	0.081
Ridge Classifier	var_smoothing	0.001
Logistic Regression	C	0.01
	Penalty	“l2”
	solver	“liblinear”

**Table 3:** Averaged internal cross-validation results.

Classifier	F2 Score	AUC	Recall	Accuracy	Cohen’s Kappa	MCC
Deep Neural Network	0.600	0.607	0.677	0.617	0.199	0.251
Random Forest	0.606	0.681	0.760	0.636	0.250	0.297
K-Nearest Neighbors	0.665	0.722	0.850	0.648	0.298	0.364
Bagging	0.619	0.709	0.732	0.696	0.320	0.352
Gradient Boosting	0.654	0.724	0.791	0.685	0.342	0.382
Support Vector Machine	0.621	0.687	0.795	0.624	0.235	0.282
Decision Tree	0.584	0.648	0.763	0.581	0.193	0.241
Naïve Bayes	0.580	0.671	0.701	0.654	0.255	0.285
Ridge Classifier	0.504	0.634	0.590	0.660	0.208	0.227
Logistic Regression	0.587	0.679	0.701	0.666	0.274	0.301

AUC: area under receiver operating characteristic curve. MCC: Matthews correlation coefficient.

**Table 4:** Performance of classification models on predicting mean heart dose in patients.

Classifier	F2 Score	AUC	Recall	Accuracy	Cohen’s Kappa	MCC
Deep Neural Network	0.789	0.818	1	0.714	0.428	0.522
Random Forest	0.775	0.803	1	0.690	0.397	0.497
K-Nearest Neighbors	0.775	0.803	1	0.690	0.397	0.497
Bagging	0.775	0.803	1	0.690	0.397	0.497
Gradient Boosting	0.762	0.787	1	0.666	0.367	0.474
Support Vector Machine	0.762	0.787	1	0.666	0.367	0.474
Decision Tree	0.725	0.742	1	0.714	0.287	0.409
Naïve Bayes	0.714	0.762	0.888	0.690	0.363	0.431
Ridge Classifier	0.714	0.762	0.888	0.690	0.363	0.431
Logistic Regression	0.701	0.747	0.888	0.666	0.333	0.406

AUC—area under receiver operating characteristic curve; MCC—Matthews correlation coefficient.

## Data Availability

The data presented in this study are available from the corresponding author upon reasonable request. Data are not available to the public due to the protection of personal information.

## Supplementary Materials

The following supporting information can be downloaded at: https://www.mdpi.com/article/10.3390/curroncol30080537/s1, File S1: Performance metrics.
