# Supplementary material for: Mean Heart Dose Prediction Using Parameters of Single-Slice Computed Tomography and Body Mass Index: Machine Learning Approach for Radiotherapy of Left-Sided Breast Cancer of Asian Patients
Source: Curr Oncol. 2023 Aug 4;30(8):7412–24. doi: 10.3390/curroncol30080537 (PMC10453557; doi:10.3390/curroncol30080537)
Supplement: Supplementary file 1 [file curroncol-30-00537-s001.zip › curroncol-2453899-supplementary.pdf]

- Accuracy:

  It measures how many observations, both positive and negative, were correctly classified.

  Accuracy = (TP+TN)/(TP+TN+FP+FN)

- Recall (TP rate):

  It measures how many observations out of all positive observations we have classified as positive. It tells us how many fraudulent transactions we recalled from all fraudulent transactions.

  Recall (TP rate) = TP/(TP+FN)

- F2 score:

  It's a metric that combines precision and recall, putting emphasis on recall.

  F2 score = TP/(TP+0.2×FP+0.8×FN)

- AUC:

  The area under a chart that visualizes the tradeoff between the true positive rate (TPR) and false positive rate (FPR), this chart is called the ROC curve.

  AUC = 0.5-FP rate/2+TP rate/2

  While FP rate equal to, FP/(FP+TN)

- Matthews correlation coefficient (MCC):

  It's a correlation between predicted classes and ground truth. The MCC is in essence a correlation coefficient value between -1 and +1. A coefficient of +1 represents a perfect prediction, 0 is an average random prediction, and -1 an inverse prediction. The statistic is also known as the Phi coefficient.

  MCC = ((TP×TN)-(FP×FN))/((TP+FP)×(TP+FN)×(TN+FP)×(TN+FN))

- Cohen’s Kappa (k):

  In simple words, Cohen Kappa tells you how much better your model is over the random classifier that is predicted based on class frequencies.

  To calculate it one needs to calculate two things: “observed agreement” (po) and “expected agreement” (pe). Observed agreement (po) is simply how our classifier predictions agree with the ground truth, which means it is just accuracy. The expected agreement (pe) is how the predictions of the random classifier that samples according to class frequencies agree with the ground truth, or accuracy of the random classifier. The kappa statistic, which is a number between -1 and 1. The maximum value means complete agreement; zero or lower means chance agreement.

$$k = (po-pe)/(1-pe)$$
